# Dirty laundry: The nature and substance of seeking relationship help from strangers online

**DOI:** 10.1177/02654075211046635

**Published:** 2021-10-23

**Authors:** Charlotte Entwistle, Andrea B. Horn, Tabea Meier, Ryan L. Boyd

**Affiliations:** 1 Department of Psychology, 4396 Lancaster University, UK; 2 Department of Psychology, 27217 University of Zurich, Switzerland; 3 University Research Priority Program: “Dynamics of Healthy Aging”, 27217 University of Zurich, Switzerland; 4 Security Lancaster, 4396 Lancaster University, UK; 5 Data Science Institute, 4396 Lancaster University, UK

**Keywords:** Relationship help-seeking, natural language analysis, relationship problems, attachment, social media

## Abstract

Interpersonal relationships are vital to our well-being. In recent years, it has become increasingly common to seek relationship help through anonymous online platforms. Accordingly, we conducted a large-scale analysis of real-world relationship help-seeking to create a descriptive overview of the nature and substance of online relationship help-seeking. By analyzing the demographic characteristics and language of relationship help-seekers on Reddit (*N* = 184,631), we establish the first-ever big data analysis of relationship help-seeking and relationship problems *in situ* among the general population. Our analyses highlight real-world relationship struggles found in the general population, extending beyond past work that is typically limited to counseling/intervention settings. We find that relationship problem estimates from our sample are closer to those found in the general population, providing a more generalized insight into the distribution and prevalence of relationship problems as compared with past work. Further, we find several meaningful associations between relationship help-seeking behavior, gender, and attachment. Notably, numerous gender differences in help-seeking and romantic attachment emerged. Our findings suggest that, contrary to more traditional contexts, men are more likely to seek help with their relationships online, are more expressive of their emotions (e.g., discussing the topic of “heartache”), and show language patterns generally consistent with more secure attachment. Our analyses highlight pathways for further exploration, providing even deeper insights into the timing, lifecycle, and moderating factors that influence *who, what, why, and how* people seek help for their interpersonal relationships.

Interpersonal relationships are vital to our well-being, yet they are complex and often difficult to navigate. The centrality of relationships to our lives is underscored by the consequences that emerge from relationship *problems*. People going through relationship difficulties report higher rates of sleep disorders (Chen et al., 2015), worse academic performance (Field et al., 2012), and mental health issues (McShall & Johnson, 2015). Perhaps unsurprisingly, romantic breakups are ranked as one of life’s most distressing events (LeFebvre et al., 2015).

When facing relationship problems, we often engage in relationship help-seeking as a means to improve our relational well-being, using other people as a resource to bring alignment between our own expectations and reality (Holmberg & MacKenzie, 2002). Today, however, we increasingly seek help for life stressors in online spaces, ranging from traditional support forums to social networking sites such as Facebook (Pan et al., 2020). This shift to online platforms provides new opportunities to study the underlying drivers of relationship help-seeking behavior at large scale in real-world contexts. Using modern natural language processing methods, we can begin to see—for the first time—a high-resolution, naturalistic view of relationship problems and relationship help-seeking behavior in the general population. In doing so, we seek to gain a “big picture” perspective on the everyday prevalence of relationship problems as they are experienced by the general public (rather than, for example, clinical/counseling samples), as well as a better understanding of *who* experiences those problems. In this article, we:

1. Provide a brief overview of the changing nature of relationship help-seeking;
2. Identify new opportunities to leverage naturalistic, online data sources to better understand people and their romantic relationships;
3. Empirically examine the characteristics, substance, and nature of relationship problems and relationship help-seeking behavior through big data analytics.

## A brief overview of the history of relationship help-seeking

**Figure 1.**
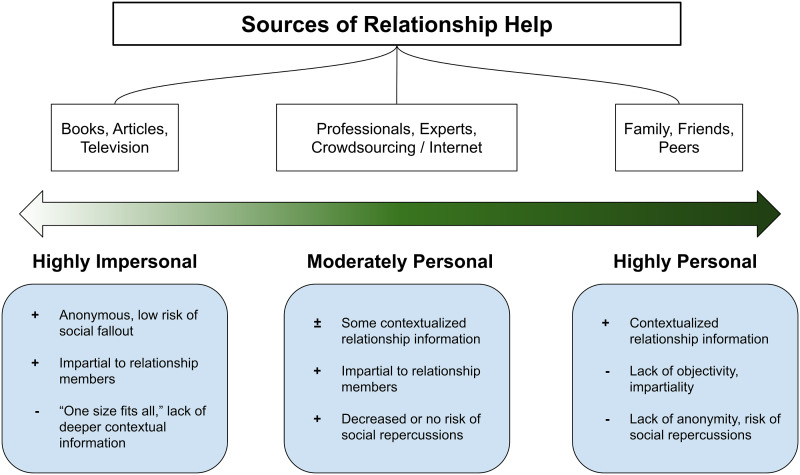
The personal–impersonal dimension in relation to sources of relationship help. *Note*. Sources by which relationship help-seeking occurs, varying in degrees of personal knowledge and connectedness to help-seekers.

Throughout history, humans have turned to others for relationship help, ranging from close acquaintances to relying on impersonal, generic truisms, and cultural norms—and each with its own benefits and drawbacks (see Figure 1). In pre-literary history, humans were necessarily limited to seeking help from those to whom they had physical access, such as members of one’s family, tribe, or geographic region. One of the benefits of help-seeking from close others surrounds shared knowledge and context, which can lead to more effective and meaningful advice-giving and receiving (Guntzviller et al., 2017). However, relationship help-seeking in personal contexts can have drawbacks as well, including a lack of objectivity or impartiality.

Historically, professional or “expert” sources have acted as a source of relationship support during times of difficulty; such support figures have often included religious authorities (e.g., Onedera, 2007), self-styled relationship gurus, and well-trained professionals. In the early 20th century, professional marriage counseling emerged from the eugenics movement (see Stone, 1949), later transforming into a fully-fledged, empirical practice (Gurman & Fraenkel, 2002). A strength of professional sources of relationship support is their often minimal personal involvement, providing a balance of objectivity and impartiality, yet affording the opportunity for some degree of personalized and context-aware feedback.

At the most impersonal extreme, people have commonly sought relationship help from static, “one-size-fits-all” resources, such as newspaper articles and books published in the popular press. The 1990s were virtually awash in relationship self-help books publications, with *Men Are from Mars, Women Are from Venus* (Gray, 1992) selling over 15 million copies to date (Beaumont-Thomas, 2017). While affording anonymity and some potential for objectivity, boilerplate relationship help sources are often too impersonal to be effective (Rosen et al., 2015).

## Relationship help-seeking in an online world

Relationship help-seeking has continued to evolve in the digital age. Online social media help us connect, create, and collaborate with people whom we have never met, making the internet a particularly appealing medium for social and informational support. For the first time in history, individuals can leverage massive communities of complete strangers for relationship help, receiving support that is personalized, information-rich, and free from the immediate social pressures created by in-person support networks. Indeed, discussing relationship problems online has become a common feature of modern relationships (Kim et al., 2017).

Often compared favorably to traditional sources of support, online spaces provide help-seekers with insights from ever-growing numbers of diverse individuals, with the added benefit of anonymity (Walther & Boyd, 2002; Wright, 2016; cf. Yip, 2020). The benefits of internet-facilitated support, and the anonymity that it provides, become particularly visible when coping with topics that are often hard to share with real-life acquaintances, such as highly intimate or stigmatized topics (Davison et al., 2000; Stephens-Davidowitz, 2017). For example, the internet is commonly used to solicit mental health support (DeAndrea, 2015). Groups who experience greater difficulties and stigma with help-seeking across domains (mental health, relationships, etc.) may be more likely to seek help through online platforms due to the anonymity that they provide (see Hammer et al., 2013; Watkins & Jefferson, 2013).

## Current study

Although past research has explored traditional relationship help-seeking among close others and within professional contexts (e.g., Doss et al., 2009; Stewart et al., 2016), there is limited research into the online relationship help-seeking process, including which types of relationship problems motivate anonymous help-seeking online in the first place. Using modern data sources and analytic methods, we can begin to explore questions surrounding the *who*, *what*, *why*, and *how* of relationship help-seeking *in situ*—that is, the real-world lived experiences of the general public.

To date, we are not aware of any research that has conducted a naturalistic, large-scale exploration of relationship problems in the general population. Accordingly, the present study aims to broadly understand the characteristics of help-seeking in the digital sphere. We additionally seek to explore how the analysis of rich, real-world language data might provide insights into the prevalence of relationship problems, as well as individual characteristics related to those problems. In particular, we aim to address three broad research questions with our work:

• RQ1: What is the demographic profile of individuals who seek relationship help online?
• RQ2: What are the central relationship problems faced today?
•RQ3: Does online help-seeking behavior provide real-world evidence for gender differences in attachment states?

### RQ1: What is the demographic profile of individuals who seek relationship help online?

The bulk of what we understand today about the psychosocial and demographic characteristics of people who seek help for relationship problems originates from research conducted in professional contexts (e.g., couples therapy). In such contexts, female partners tend to recognize their relationship problems and actively seek professional relationship help more than male partners (for a review, see Stewart et al., 2016). The decision to seek professional relationship help is additionally influenced by age, with middle-adulthood couples being more likely to actively seek professional relationship help (Doss et al., 2003). Specifically, average age ranges are usually in the realm of 38–41 years for those couples that typically seek professional help (Duncan et al., 2020; Schofield et al., 2015).

Whether the demographics of individuals who anonymously crowdsource relationship help in online spaces match those of people who typically seek professional relationship help is unknown. Valuably, this knowledge should allow for greater understanding of the facilitators and barriers to seeking help for relationship problems. If online help-seekers are primarily middle-aged women, as in professional contexts, we may speculate that online platforms simply provide an alternative, less resource-consuming option. Should online relationship help-seekers show divergent demographics, however, we may suggest that online spaces provide a support platform for individuals who traditionally would not have sought relationship help from others due to well-established treatment barriers, such as stigma, time, and financial cost (see, e.g., Hall & Sandberg, 2012; Werner-Wilson & Winter, 2010; Williamson et al., 2019). In our study, we create an initial, descriptive understanding of online relationship help-seekers through basic demographic characteristics to which we have immediate access, namely, age and gender.

### RQ2: What are the central relationship problems faced today?

As with the question of *who* seeks help for their relationship problems, our understanding of *what* relationship problems motivate people to seek help are largely based on research in professional contexts. For example, communication difficulties are often cited as the most common motivator for seeking professional relationship help. Other leading reasons typically include issues with physical and emotional intimacy, trust, finances, and housework, to name a few (see: Doss et al., 2004; Duncan et al., 2020; Roddy et al., 2019; Schofield et al., 2015). Given the extreme differences in prequisites for seeking relationship help professionally versus online, it could be expected that the main motivations for seeking relationship help differ between these support contexts. Put another way, past research on relationship problems in the context of formal interventions (both online and in-person; e.g., Roddy et al., 2019) are critical, but likely reflect skewed representations of relationship problem distributions and prevalence in the general public, and in everyday life. For example, ∼ 1% of couples raise “abuse” as a relationship problem in intervention settings (Roddy et al., 2019), whereas the CDC reports between 5–6% of the general US population has experienced some form of intimate partner abuse or violence within the past 12 months (Basile et al., 2011). Such discrepancies highlight serious under-representation of prevalent relationship problems in professional settings.

Similarly, discrepancies in gender distributions of relationship problems may be reflected differently in the general public relative to intervention settings. Many problems are reported fairly equally by both men and women (Duncan et al., 2020), however, some gender differences do exist. Relative to men, women are more likely to report partner-specific traits and behaviors as problematic, and men report problems with physical intimacy more than women (e.g., Roddy et al., 2019). As noted earlier, it could reasonably be argued that such differences may result, in part, from social pressures arising from stereotypes and gender norm expectations. Put another way, if existing gender differences are (at least partially) a product of stigmatization and pressure to conform to gender stereotypes (e.g., Cheng et al., 2015), we may expect diminished, or at least different, gender differences in relationship problems shared in anonymous contexts.

Accordingly, in the present study, we explore the psychosocial topography of relationship problems as they are discussed by online help-seekers. We employ modern text analysis methods—namely, the Meaning Extraction Method (MEM; Chung & Pennebaker, 2008)—as a way to create a high-level map of the most common relationship problems discussed online. Briefly described, the MEM is a topic modeling technique that extracts psychologically meaningful themes from natural language—this process works by identifying clusters of words that frequently co-occur across a text corpus. The MEM has demonstrated value for understanding the psychosocial dynamics of online communities (Blackburn et al., 2018; Currin-McCulloch et al., 2021). We investigate potential gender differences in the topics raised by the online help-seekers insofar as individuals from each gender divulge various relationship problems.

### RQ3: Does online help-seeking behavior provide real-world evidence for gender differences in attachment states?

There is clear consensus that a person’s attachment style—characterized by the mental models of the self and social bonds over the lifespan—is essential to close relationships, manifesting in the form of discrete attachment *states* and relationship behaviors and cognitions (Hazan & Shaver, 1987; Mikulincer & Shaver, 2016). Importantly, gender differences in attachment have been reported (Scharfe & Bartholomew, 1994; Schmitt, 2003), with recent work suggesting that, in general, men are more prone to dismissive attachment and are less emotionally invested, whereas women are more emotionally invested and prone to preoccupied attachment (Haydon et al., 2014) and/or secure attachment (Grabill & Kerns, 2000). However, the question of whether persistent gender differences exist in attachment is far from resolved (Bakermans-Kranenburg & van IJzendoorn, 2009), particularly in real-world and everyday life. In our goal to better understand the “why” and “how” of relationship help-seeking, we were motivated to explore gender differences through the lens of romantic attachment in the real-world.

Relationship help-seeking is a salient and emergent process of attachment states, and the ability to passively examine gender differences in romantic attachment via digital traces helps to shed light on the nature and development of gender-differentiated behavior in the context of romantic relationships—a key domain with often contentious and conflicting findings. As with the previous research questions, we note that other social factors, such as real or perceived pressure to conform to gender stereotypes, may be a driving force in shaping how attachment states manifest (see Pauletti et al., 2016). Here too, a real-world analysis of attachment states should provide insight into whether stereotypic attachment states are largely a reflection of immediate social pressures or, alternatively, that attachment states are consistent with more general findings of long-term attachment styles (see Del Giudice, 2019).

Importantly, attachment itself is observable in verbal behavior when discussing one’s relationships (Horn & Meier, in press). Using an established language analysis program, Linguistic Inquiry and Word Count (LIWC; Pennebaker et al., 2015), we quantify relevant language variables from relationship help solicitations. Briefly described, LIWC is a text analysis program that relies on an internal dictionary to map words to psychologically meaningful categories. The psychometric validity of LIWC been extensively demonstrated across thousands of studies in disciplines as diverse as psychology, computer science, and communication (Tausczik & Pennebaker, 2010).

To date, very few studies have explicitly explored gender differences in verbal behavioral markers of attachment. In the current study, we significantly expand on past work both in terms of sample size and variable scope by examining a number of additional language categories that can be reasonably expected to reflect attachment states, including a wider range of emotions (rather than solely focusing on anger), cognitive processes, and affiliation (see Table 1). Additional categories were selected on the basis of their theoretical relevance to expand the limited nomological network of associations between attachment, gender, and verbal behavior.

**Table 1.** Language measures included in the current study and their previously reported relationships to attachment states.

Language measure	Example words	Attachment state	Reference(s)
Word count	N/A	(−) Dismissive (+) Preoccupied	Waters et al. (2016) Cassidy et al. (2012) O’Hara (2007; cited from Waters et al., 2016)
I-words	I, me, my	(+) Preoccupied	Dunlop et al. (2020)
We-words	We, us, our	(+) Secure attachment (−) Insecure attachment	Dunlop et al. (2020) Borelli et al. (2019)
Negations	No, not, didn’t	(−) Secure attachment (+) Insecure attachment	Waters et al. (2016) Cassidy et al. (2012)
Prepositions	After, near, close	(−) Dismissive	Waters et al. (2016)
Conjunctions	But, also, and	(−) Dismissive	Waters et al. (2016)
Tentative language	Might, could, maybe	(+) Dismissive	Waters et al. (2016)
Filler words	Like, so, erm	(−) Dismissive	Waters et al. (2016)
Anger words	Angry, furious, mad	(+) Preoccupied	Borelli et al. (2013) Cassidy et al. (2012) Waters et al. (2016)
Negative emotion	Sad, angry, anxious	(+) Preoccupied	*Novel prediction*
Positive emotion	Happy, excited, joy	(+) Secure attachment	*Novel prediction*
Sadness	Depressed, tearful, upset	(+) Preoccupied	*Novel prediction*
Anxiety	Anxious, scared, worry	(+) Preoccupied	*Novel prediction*
Cognitive processes	Think, puzzle, solve	(+) Preoccupied	*Novel prediction*
Affiliation	Together, social, collectively	(+) Secure attachment	*Novel prediction*
Absolutism	Always, never, definitely	(+) Preoccupied	*Novel prediction*

Here, we briefly highlight our rationale for the inclusion of each of the additional language variables. First, affiliation reflects positive engagement/connectedness with others; individuals who are more securely attached have been found to attach high importance to affiliation goals in friendship (Mikulincer & Selinger, 2001) and can be expected to likely think to a greater degree along an affiliative dimension when discussing relationships (for an in-depth discussion on the relationship between psychological dimensions and verbal behavior, see Boyd & Pennebaker, 2017; Boyd & Schwartz, 2021). Relatedly, one might anticipate that the broad expression of negative emotions in the context of relationship discussions would be associated with preoccupied attachment, based on the definition and characteristics of such attachment state (i.e., being *anxiously* attached), whereas dismissive individuals tend to rely on less emotionally immediate language (Borelli et al., 2013). Conversely, it could be intuitively presumed that people who are securely attached would express more positive emotion and less negative emotion when discussing relationships.

More broadly, “cognitive processing” language reflects greater cognitive load, “working through” a problem, or preoccupation, such as is seen following a traumatic event or relationship difficulty (e.g., D’Andrea et al., 2012). Individuals with a preoccupied attachment style should therefore use relatively greater cognitive processing language when discussing their romantic relationships.^1^ Last, a greater “all or nothing” type of thinking may be indicative of preoccupied attachment, given that high rates of absolutist language have also been associated with problematic patterns of affect (see Al-Mosaiwi & Johnstone, 2018).

## Methods

### Data collection

For all research questions, we analyzed a large collection of submissions to Reddit, one of the most frequently visited websites on the planet (Alexa, 2020). Briefly described, Reddit is a massive, anonymous online discussion forum composed of thousands of sub-forums (i.e., “subreddits”), each founded around specific topics (e.g., musicians, cooking, etc.). Within each subreddit, users can create threads (i.e., “submissions”) about a particular topic or respond to one another through hierarchically structured “comments.” As Reddit is anonymous, publicly accessible, and content rich, it poses as a rich source of social psychological natural language data.

We explored data from the *r/relationships* subreddit, one of the largest online communities for relationship help-seeking, comprising over three million members. *r/relationships* is self-described as:

“...a community built around helping people and the goal of providing a platform for interpersonal relationship advice between redditors. We seek posts from users who have specific and personal relationship quandaries that other redditors can help them try to solve.”

Data were extracted from the larger PushShift database (Baumgartner et al., 2020) using a custom-made Python pipeline. Given that the focus of the present research is on relationship help-seeking (as opposed to the provision of relationship help), we collected only submissions made by users and not comments in response to submissions. Only users with a single submission to the *r/relationships* subreddit were collected, ensuring data independence and preventing over-representation of high-activity users. Submissions were collected across the full lifetime of the subreddit, spanning approximately 12 years (*N* = 521,536).

### Data pre-processing and preparation

Data collected from the *r/relationships* subreddit were cleaned and prepared for analysis according to standard guidelines (Boyd, 2017): formatting errors were corrected, HTML entities converted to American Standard Code for Information Interchange (ASCII), and texts containing fewer than 25 words were omitted. Given our interest in exploring explicitly romantic relationships, we only retained submissions that were categorized by users (through “flairs” attached to posts; see Supplementary Materials A) as related to romantic relationships, which included the “relationships,” “dating,” “break-ups,” and “infidelity” categories. Pre-processing resulted in 184,631 submissions being retained from the same number of unique users.

Reddit is an anonymous platform, and demographic data is not usually available for individual users. In the *r/relationships* subreddit, however, submitters typically disclose their age and gender, as well as the age and gender of their relationship partner(s), within the title of their submission. For example, a 36-year-old man discussing a relationship problem they are having with their 34-year-old female spouse may provide contextual clues by writing “I [36/M] and my wife [34/F]....” This unique feature allowed us to automatically extract age and gender data for a majority sample users via regular expressions tailored specifically to the current dataset (for a recent, similar example, see Jagfeld et al., 2021).^2^ In total, we were able to extract demographic data for 80.05% (*N* = 147,796) of the users within our sample. Note that for the sake of simplicity, we use the term “romantic partner” to refer to the relationship partner(s) being discussed, including current, past, or speculative partners.

## Results

### RQ1: What is the demographic profile of individuals who seek relationship help online?

To understand the demographic composition of individuals seeking relationship help online, we examined age and gender compositions of *r/relationships* users who provided such information, along with their romantic partners’ age/gender composition (see Table 2). Additional analyses of user gender by submission flair frequencies are presented in Supplementary Materials A.

**Table 2.** Age and gender composition of r/relationships users and their romantic partners.

		*r/relationships* users			Romantic partners		
		Total *N* (%)	Men *N* (%)	Women *N* (%)	Total *N* (%)	Men *N* (%)	Women *N* (%)
Gender		147,796	80,722 (54.62%)	67,074 (45.38%)	130,404	58,692 (45.01%)	71,712 (54.99%)
Age		147,795	80,722 (54.62%)	67,073 (45.38%)	130,398	58,689 (45.01%)	71,709 (54.99%)
	<12 years old	1 (0.00%)	1 (0.00%)	0 (0.00%)	9 (0.01%)	5 (0.01%)	4 (0.01%)
	12–17 years old	7275 (4.92%)	5447 (6.75%)	1828 (2.73%)	6762 (5.19%)	1186 (2.02%)	5576 (7.78%)
	18–24 years old	81,208 (54.95%)	43,387 (53.75%)	37,821 (56.39%)	66,150 (50.73%)	23,998 (40.89%)	42,152 (58.78%)
	25–34 years old	53,928 (36.49%)	28,741 (35.60%)	25,187 (37.55%)	49,760 (38.16%)	28,470 (48.51%)	21,290 (29.69%)
	35–44 years old	4765 (3.22%)	2776 (3.44%)	1989 (2.97%)	6386 (4.90%)	4198 (7.15%)	2188 (3.05%)
	45–54 years old	562 (0.38%)	340 (0.42%)	222 (0.33%)	1022 (0.78%)	645 (1.10%)	377 (0.53%)
	55–64 years old	51 (0.03%)	28 (0.03%)	23 (0.03%)	268 (0.21%)	167 (0.28%)	101 (0.14%)
	65 years or older	5 (0.00%)	2 (0.00%)	3 (0.00%)	41 (0.03%)	20 (0.03%)	21 (0.03%)
	Mean (*SD*)	24.04 (5.00)	23.90 (5.25)	24.22 (4.68)	24.66 (5.87)	26.28 (5.99)	23.33 (5.41)

One striking pattern in gender distributions is that, contrary to what is commonly found in professional settings, more men solicited relationship help through *r/relationships* than women, with 54.62% of the users being men, and only 45.38% being women. Among users’ romantic partners, the relative gender composition is almost directly reversed, with 45.01% being men and 54.99% being women, reflecting that the majority of the sample consisted of mixed-gender relationships (95.53%).

The mean age of online relationship help-seekers (24.04 years) was considerably younger than average age ranges typically found in professional contexts (i.e., 38–41 years; Duncan et al., 2020; Schofield et al., 2015), with the majority of users falling in the 18–24 age bracket (54.95%). There was a small, statistically significant difference in the age of men and women seeking relationship help online, with women being slightly older (*t* = −12.17; *p* < .001; *d* = .06).

There were interesting and distinctive trends in gender-by-age composition in our sample, such as there being considerably more adolescent boys (*N* = 5447) than girls (*N* = 1828) seeking help. Although our exploration of demographic characteristics provides a novel glance into who seeks relationship help online, we note that these findings may also simply mirror the more general composition of Reddit, which skews toward young males (Duggan & Smith, 2013).

### RQ2: What are the central relationship problems faced today?

To explore the topography of relationship problems within our sample—both in terms of content and distribution—we analyzed *r/relationships* user’s solicitations for relationship help using the MEM (Chung & Pennebaker, 2008; for additional discussions of the MEM, see also: Boyd & Pennebaker, 2016; Markowitz, 2020). For MEM analyses, we used BUTTER (Boyd, 2020), an open-source text analysis application for social scientists. The MEM conducted on *r/relationships* submissions resulted in 25 themes that reflected the most prevalent relationship problems. Table 3 presents both the content and distribution of each MEM theme. Briefly described: when considering the *Mean* column, we see the relative importance of each theme insofar as the typical amount that it is discussed in any given submission. More importantly, however, is the *Frequency* column, which describes the number of submissions that invoked each theme (for examples and information on themes and theme extraction, see Supplementary Materials C). Note that any particular submission may contain multiple themes, for example, sexual problems *and* communication issues. Tellingly, the mean number of themes present in any given *r/relationships* submission was 2.81 (median = 3; *SD =* 1.92), highlighting that the majority of submissions (>50%) were made by users who were motivated to seek help not for a single relationship problem, but rather larger constellations of problems.

**Table 3.** Content and distribution of themes extracted by the MEM on r/relationships submissions, in order of mean percentage of discussions (*N* = 184,631).

Theme	Example words	Mean (*SD*)	Frequency (% of sample)
Heartache	Heart, break, hurt	14.59 (2.96)	37836 (20.13%)
Communication	Discuss, express, conversation	11.37 (2.64)	34026 (18.10%)
Shared feelings	Told, upset, feeling	10.62 (2.43)	35709 (19.00%)
Time	Morning, Friday, hour	6.68 (2.50)	27435 (14.60%)
Dating	Date, casual, hook-up	6.00 (2.60)	28619 (15.23%)
Personal qualities	Cool, nice, funny	5.20 (1.98)	25255 (13.44%)
Trust issues	Trust, snoop, cheat	4.55 (2.34)	23201 (12.34%)
Intimacy	Smile, cuddle, touch	3.93 (1.92)	22671 (12.06%)
Partying	Party, drunk, Invite	3.49 (1.77)	23145 (12.31%)
Abuse	Abusive, threaten, control	3.34 (1.67)	22822 (12.14%)
Distance	Move, travel, long-Distance	3.00 (1.83)	19828 (10.55%)
Wedding	Marriage, marry, wedding	2.78 (1.41)	22767 (12.11%)
Career	Job, career, company	1.78 (1.51)	14337 (7.63%)
Finances	Money, pay, debt	1.63 (1.61)	9994 (5.32%)
Family/Parenting	Child, pregnancy, parent	1.58 (1.51)	13391 (7.12%)
Mental health issues	Depression, diagnose, therapy	1.29 (1.10)	14950 (7.95%)
School	School, college, semester	1.08 (1.56)	24665 (13.12%)
Hobbies	Video game, music, sport	0.85 (1.34)	28763 (15.30%)
Religion	Religious, belief, church	0.67 (0.94)	8442 (4.49%)
Housework	Cleaning, laundry, cooking	0.60 (0.84)	6142 (3.27%)
Sex	Sex, masturbate, porn	0.56 (1.22)	10117 (5.38%)
Language	English, native, language	0.55 (1.00)	21122 (11.24%)
Body weight	Lose-weight, overweight, diet	0.40 (0.68)	2259 (1.20%)
Substance use	Drinking, drug, addict	0.18 (0.44)	2459 (1.31%)
Romantic gestures	Thoughtful, gift, celebrate	0.01 (0.81)	48767 (25.95%)

*Note.* MEM = Meaning Extraction Method

*Note.* This table describes the content and distribution of the 25 themes generated from the MEM on *r/relationships* submissions. The mean values represent the mean percentage by which each theme was discussed relative to the entirety of *r/relationships* discussions. The frequency values represent the number and percentage of submissions that mentioned each theme (i.e., whereby the theme was present).

**Figure 2.**
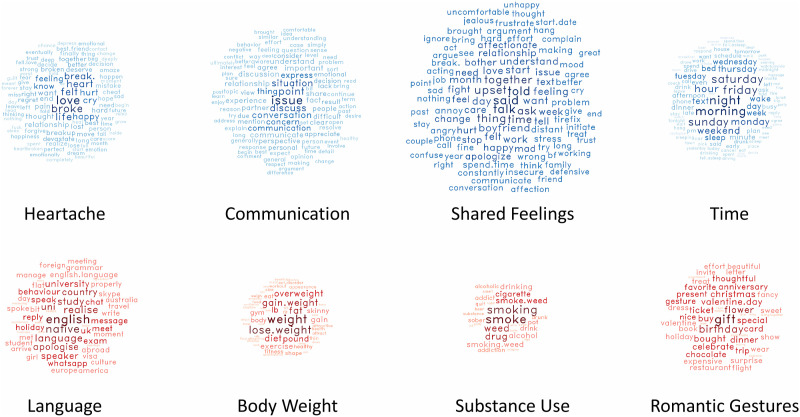
The four most-discussed (top row, blue) and least-discussed (bottom row, red) relationship problem themes.

Consistent with past studies of professional relationship help-seeking, communication was a central motivator for help-seeking in our sample, with the second and third most commonly discussed topics relating to communication. Notably, “heartache” was the most commonly discussed theme, indicating the psychological distress caused by the relationship problems being discussed. Other frequently discussed themes included: focus on time, casual dating, personal qualities, trust issues, intimacy, partying, and abuse. Romantic gestures, substance use, and body weight were considerably less common (see Supplementary Materials C for additional notes). Visualizations of the four top- and bottom-scoring MEM themes, by mean, are illustrated in Figure 2.

**Figure 3.**
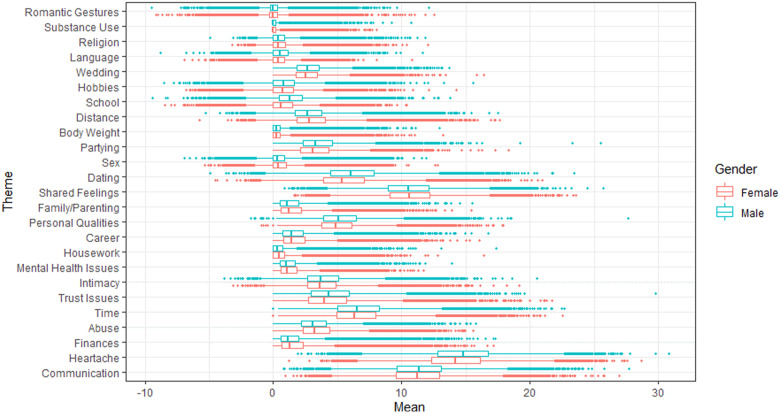
Boxplots showing mean percentages of Meaning Extraction Method themes split by gender of user (N = 147,796). *Note.* The full table of statistical comparisons for each theme is presented in Supplementary Materials D.

We performed additional analyses comparing men and women’s use of each MEM by comparing mean percentages (see Figure 3; full analyses presented in Supplementary Materials D). Most gender differences found were generally small, but theoretically meaningful. The largest gender difference was the use of the “school” theme, with men spending more time discussing things related to school than women—a potential byproduct of the age difference within our sample. More pronounced and meaningful gender differences emerged, with men more commonly discussing themes of heartache, dating, partying, personal qualities, and language; women spent more time discussing themes related to finances, abuse, distance, and housework.

### RQ3: Does online help-seeking behavior provide real-world evidence for gender differences in attachment states?

To examine the relationship between gender and romantic attachment, we conducted independent-samples *t*-tests on each LIWC metric presented in Table 1, using user gender as the predictor. Results are presented in Table 4, with visual presentation in Supplementary Materials B.

**Table 4.** Gender differences in language categories indicative of romantic attachment states (*N* = 147,796).

Language category	Mean (*SD*)		*t*	*d*	95% CI
	Men (*N* = 80,722)	Women (*N* = 67,074)			
Word count	524.17 (402.93)	555.74 (375.96)	−15.55***	.08	−35.54 to −27.59
I-words	8.13 (2.23)	8.44 (2.23)	−26.68***	.14	−.33 to −.29
We-words	1.72 (1.20)	1.64 (1.15)	14.60***	.07	.08–.10
Cognitive processes	14.44 (2.91)	14.48 (2.79)	−3.02**	.01	−.07 to −.02
Conjunctions	7.93 (1.55)	8.13 (1.47)	−26.07***	.13	−.22 to −.19
Prepositions	13.68 (2.01)	13.33 (1.89)	34.59***	.17	.33–.37
Filler words	0.05 (0.14)	0.05 (0.13)	3.04**	.02	.00–.00
Affiliation	4.94 (2.01)	4.80 (2.02)	13.72***	.07	.12–.16
Positive emotion	3.00 (1.28)	2.96 (1.29)	5.36***	.03	.02–.05
Negative emotion	2.48 (1.30)	2.69 (1.35)	−29.39***	.16	−.22 to −.19
Anger	0.67 (0.69)	0.75 (0.73)	−22.00***	.11	−.09 to −.07
Sadness	0.58 (0.58)	0.57 (0.57)	.17	.02	−.01–.01
Anxiety	0.53 (0.54)	0.61 (0.56)	−27.34***	.15	−.08 to −.07
Negations	2.41 (0.96)	2.53 (0.93)	−23.86***	.13	−.13 to −.11
Tentativeness	3.29 (1.37)	3.20 (1.29)	13.55***	.07	.08–.11
Absolutism	0.97 (0.60)	1.03 (0.59)	−17.11***	.10	−.06 to −.05

**p<.01, ***p<.001.

*Note*. Means refer to percentages of the total words used. CI = confidence interval.

Our results indicated a clear, patterned association between gender and linguistic markers of attachment. When discussing their relationships, women (relative to men) used language consistent with more of a preoccupied attachment state (consistent with prior research findings and expectations; see Table 1), with greater words overall used, more self-focused language (i.e., I-words), cognitive processing language, negations, absolutist language, overall negative emotion, anger, and anxiety words; this pattern was matched by less couple-focused language (i.e., we-words), affiliative language, and positive emotion words. Contrastingly, men showed language patterns more consistent with a secure attachment state: greater use of we-words, affiliation words, and positive emotion words, paired with lower rates of I-words, cognitive processing words, negations, absolutist language, and overall negative emotion, anger, and anxiety words. However, some patterns indicative of dismissive attachment were present among men (relative to women) including fewer words used overall, fewer prepositions, fewer filler words, and more tentative language.

## Discussion

In the present study, we provide novel insights into the nature and substance of relationship problems—based on a sample of Reddit users—using natural language analysis methods. To our knowledge, this is the first study that has provided a large-scale, high-resolution, naturalistic view of relationship problems and relationship help-seeking *in situ* within the general population.

The first aim of the present study was to describe the demographic composition of online relationship help-seekers relative to those who typically seek help in more traditional/professional contexts. We examined the age and gender of individuals seeking relationship help online via the *r/relationships* subreddit, finding a greater percentage of men soliciting relationship help than women. Interestingly, this differs from traditional, professional contexts, where women are typically more willing and active in seeking help for their relationship problems compared to male partners (Stewart et al., 2016). This discrepancy in findings supports our notion that men may find anonymous, online relationship help settings preferable to in-person contexts, likely due to stigma attached to help-seeking behavior in men (Hammer et al., 2013; Vogel et al., 2011). As mentioned above, these results could also be interpreted as an over-representation of help-seeking by female users relative to the baseline demographic composition of our sample (Duggan & Smith, 2013). Given that we do not have access to the demographics of passive users who do not post to the subreddit, we suggest that our conclusions on the contribution of gender toward the propensity to seek relationship help online be interpreted tentatively.

Those posting to the *r/relationships* platform were found to be considerably younger (average age 24 years) than people who typically seek relationship help in more traditional contexts (average age range 38–41 years; Duncan et al., 2020; Schofield et al., 2015), with the majority of *r/relationships* users falling in the 18–24 age bracket. This finding suggests that the anonymous, convenient, and broadly accessible nature of the online help-seeking space enables those who traditionally under-represented or less likely to seek help (e.g., young men) by overcoming barriers related to stigma or resource availability. These results complement the wider support-seeking literature highlighting that online spaces provide greater opportunities for support-seeking through the erosion of barriers associated with traditional contexts (DeAndrea, 2015; Vitak & Ellison, 2013). Notably, given that online relationship help-seeking is particularly common among younger age groups, it could be inferred that the informality of the online help-seeking environment is providing means for people to seek help and advice for more casual and early-stage relationships (e.g., at the “dating stage”) compared to the stage at which people more commonly seek professional relationship help (i.e., after several years of marriage).

Our topic modeling approach revealed 25 themes that help to illuminate the topography of relationship problems in the general public. Analysis of the distribution of themes revealed that the most commonly discussed topic on the *r/relationships* platform was “heartache,” supporting the notion that romantic dissolution and breakups are particularly distressing life events (LeFebvre et al., 2015). Moreover, the frequent discussion of feeling heartache is interesting given that this is not a specific relationship *problem* being discussed. Rather, people appear to simply be using the online platform to express their distress and seek general emotional support from others, suggesting that the emotional pain experienced following relationship problems or dissolution is perhaps the strongest motivator of reaching out for social support—more so than seeking to resolve any particular problem in and of itself.

What is particularly revealing from our analyses is that the main motivators identified for relationship help-seeking in the digital space were generally consistent with the main reasons for seeking relationships help identified from previous research in more traditional, professional contexts. Specifically, in line with previous research highlighting communication difficulty as the most common motivator for seeking professional relationship help (Doss et al., 2004; Duncan et al., 2020; Roddy et al., 2019), as well as being the leading cause for romantic breakups (Morris et al., 2015), communication was also found to be the most-discussed relationship *problem* within our sample (discounting the general *topic* of heartache). Other core themes captured from the *r/relationships* discussions are also consistent with the main reasons for professional relationship help-seeking, such as issues relating to intimacy, trust, finances, and housework. This consistency in relationship help-seeking motivators between anonymous, online contexts and more traditional, professional contexts strengthens the idea that many relationship problems are common and ubiquitous.

Critically, we find that in many cases, our results reflect more realistic real-world prevalences of relationship problems outside of therapeutic contexts. For example, the WHO reports that around 13% of surveyed women report some form of intimate partner abuse in the previous 12 months (World Health Organization, 2021); our analyses found that 12.14% of submissions contained a non-negligible reference to the “abuse” MEM theme, strongly contrasting with only 1.3% in intervention contexts (Roddy et al., 2019). Similarly, other relationship problems, such as communication difficulties and conflict, may be over-represented in traditional contexts (e.g., 27.2% in Roddy et al., 2019; our sample: 18%). Other themes showed strong convergence with past work. For example, we found highly similar rates of family/parenting problems being raised as reported in past work (7.12% in our sample; 6.6% in Roddy et al., 2019).

Our analysis of relationship problems revealed small, consistent gender differences. Among the more pronounced gender differences, men more commonly discussed themes of school (the largest gender difference), heartache, dating, partying, personal qualities, and language; women more commonly discussed themes related to finances, abuse, physical distance, and housework. Notably, the fact that the heartache theme was more commonly discussed by men emphasizes how men are at least as equally as affected by relationship problems as women and feel comfortable to express and seek support for their distress in online, anonymous settings. We therefore re-emphasize that existing gender differences identified within traditional contexts may at least partially be a result of stigmatization and pressure to conform to stereotypes. However, our finding that women discussed things like abuse, finances, and housework more than men instead indicates some continuation of gender norms “spilling over” into the online platform. Rather than eliminating or reversing gender norms, the anonymous online platform instead appears to provide a space where gender norms and stereotypes are relaxed, particularly those that carry strong stigma (e.g., expression of emotional distress by men).

Last, we explored the use of online relationship help-seeking as a digital trace for generating novel insights into the relationship between gender and romantic attachment. We examined gender differences in romantic attachment through the analysis of pre-selected linguistic markers of attachment states-of-mind, building on limited previous work in this domain. Overall, the general patterns of language used by men and women discussing their relationships on the *r/relationships* platform appears to suggest that women may be more prone to preoccupied attachment states, whereas men may be more inclined toward secure attachment states. These findings align, in part, with those from previous research suggesting that women are more prone to preoccupied attachment (Haydon et al., 2014)—and, importantly, extends them into everyday life in the real world. However, our findings run counter to previous research indicating that men are more prone to dismissive attachment (Haydon et al., 2014). While several explanations for such patterns are possible, we suggest that modern, online help-seeking platforms may allow men to behave in ways that contradict the dismissive stereotype, again highlighting the powerful role of stereotypes in in-person relationship help-seeking behavior (as similarly shown when considering cross-cultural differences; (Schmitt, 2003). Nevertheless, it is important to emphasize that we did not possess established measures of attachment style in our study. Moreover, we do not know the extent to which various attachment styles self-selected into the *r/relationships* platform, potentially skewing the representativeness of our sample.

### Limitations and future directions

While the current study comprises a large, real-world sample, it is not a globally representative sample. Given that our data were collected from a single website—albeit one of the most visited websites in the world (Alexa, 2020)—our sample may be biased in ways consistent with its user base, both demographically (e.g., younger, male, American) and psychosocially. It is therefore possible, for example, that the skew toward men and younger people within our sample could simply be a product of the demographic composition of Reddit. Despite such limitations, our sample is large, diverse, and highly international, creating a strong starting and comparison point for future research in this domain.

We also note the tentative nature of our findings pending further exploration in samples with more varied measures. For instance, within our sample, we cannot say whether gender differences were confounded with the current “stage” of relationship problems people were experiencing. Indeed, the choice to seek help online versus professionally is likely shaped by complex interactions between characteristics of the individual, such as gender and age, and characteristics of the relationships, including specific relationship problems and stage of relationship, and the language that partners use to convey and make sense of those problems. While such intricacies are beyond the scope of the current study, future research should aspire to disentangle such complexities.

Regarding our findings involving various gender differences, it is possible that women are more likely to seek relationship help once their relationship problems are at a more severe stage (see, e.g., Ansara & Hindin, 2010), whereas men may be more likely to seek relationship help at a much earlier, less severe stage, for example. Indeed, gender differences in the themes discussed do seem to suggest that men may in fact be seeking support for relatively more casual, early-stage relationship problems compared to women. For example, men more commonly discussed lighter topics stereotypically associated with youth and greater immaturity, such as dating and partying, whereas women spent more time discussing more serious topics, such as abuse and finances. Were there gender differences in the stage of relationship problems for which people were soliciting help, it is possible that this may have at least partially driven our associations found between gender and attachment state. We are unable to determine the presence or absence of such effects within our current sample.

Last, although the present findings provide novel insights into relationship help-seeking in online anonymous contexts, the quality of the help and advice given within these contexts remains unaddressed. Although the anonymous and effortless nature of the online space indeed provides numerous benefits to help-seekers, we do not know whether the advice provided in such settings is of sufficient quality to facilitate healthier relationships. If the advice provided is of poor quality, relationship problems may be exacerbated, contributing to further interpersonal problems. We anticipate further analyses of anonymous, online relationship discussion platforms to determine the quality and subsequent implications of such advice.

## Conclusion

The present study is the first to leverage big data and modern natural language analysis techniques to better understand relationship help-seeking in naturalistic contexts in the general population. We are optimistic that future research will be able to further improve and refine upon our analyses, providing even deeper insights into the timing, lifecycle, and moderating factors that influence when, where, why, and how people seek help for their interpersonal relationships. With the expansion of AI and automated natural language generation, we expect that the near future holds high promise for increasingly useful identification of—and help with—relationship problems in everyday life.

## Supplemental Material

sj-pdf-1-spr-10.1177_02654075211046635 – Supplemental Material for Dirty laundry: The nature and substance of seeking relationship help from strangers online Click here for additional data file. Supplemental Material, sj-pdf-1-spr-10.1177_02654075211046635 for Dirty laundry: The nature and substance of seeking relationship help from strangers online by Charlotte Entwistle, Andrea B. Horn, Tabea Meier and Ryan L. Boyd in Journal of Social and Personal Relationships

## Notes

1 Directly relevant to the current study, recent findings show that increases in cognitive processing language predict impending romantic breakups (Seraj et al., 2021).

2 See https://osf.io/5qzcs for code and data.
