# Supplementary material for: Dirty laundry: The nature and substance of seeking relationship help from strangers online
Source: J Soc Pers Relat. 2021 Oct 23;38(12):3472–96. doi: 10.1177/02654075211046635 (PMC8669208; doi:10.1177/02654075211046635)
Supplement: sj-pdf-1-spr-10.1177_02654075211046635 – Supplemental Material for Dirty laundry: The nature and substance of seeking relationship help from strangers online [file sj-pdf-1-spr-10.1177_02654075211046635.pdf]

Supplementary Material for:

Dirty Laundry: The Nature and Substance of Seeking Relationship Help from Strangers Online

Charlotte Entwistle[1], Andrea B. Horn[2,3], Tabea Meier[2,3], Ryan L. Boyd[1,4,5]

[1] Department of Psychology, Lancaster University, United Kingdom

[2] Department of Psychology, University of Zurich, Switzerland

[3] University Research Priority Program: "Dynamics of Healthy Aging", University of Zurich, Switzerland

[4] Security Lancaster, Lancaster University, United Kingdom

[5] Data Science Institute, Lancaster University, United Kingdom

Author Notes

Charlotte Entwistle https://orcid.org/0000-0002-2739-2644

Andrea B. Horn https://orcid.org/0000-0003-2729-7062

Tabea Meier https://orcid.org/0000-0003-2902-4113

Ryan L. Boyd https://orcid.org/0000-0002-1876-6050

Supplementary Materials A:

An Analysis of Gender versus Submission Flair

We conducted additional, descriptive analyses to compare the frequencies of the various submission categories assigned to *r/relationships* submissions between male and female users. These categories are also known as “flairs” in the context of Reddit and, within *r/relationships*, are used to label each user submission as pertinent to a specific topic. For example, a user submission that has been assigned an “infidelity” flair signals that the submission content is primarily about infidelity within one’s relationship.

Figure S1 shows how there were generally few/small gender differences in the general relationship topics discussed — among both men and women, the general “relationships” flair was by far the most frequently assigned to submissions, this was followed by the “break-ups” and “dating” flairs, which were assigned at fairly equal rates, and finally, the “infidelity” flair was the least frequently assigned to submissions. The largest gender differences were in relation to the dating and break-ups categories, with men seeking relationship help for dating and break-ups more than women.

Figure S1

Frequency of Flairs Assigned to r/relationships Submissions Split by Gender of User (N = 147,796).

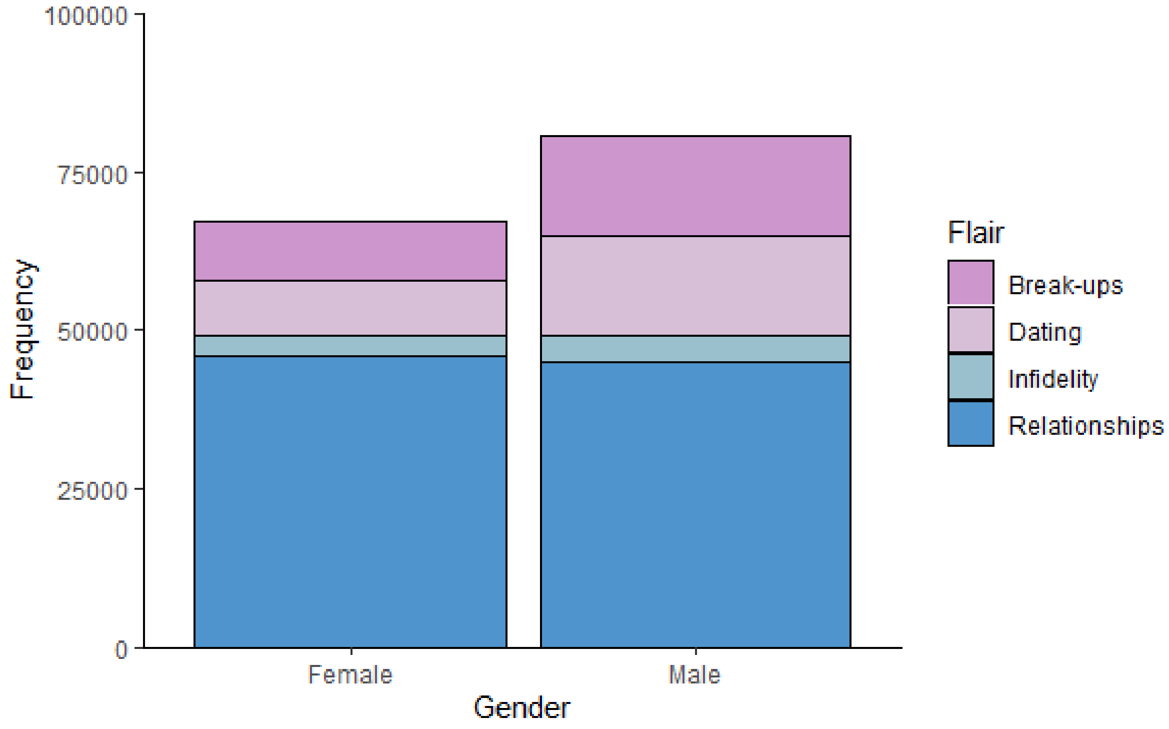

Supplementary Materials B:

Relative Frequencies of LIWC Scores by Gender, Visualized

In order to get a clearer visual sense of gender differences in language use from a LIWC perspective, we present below a figure of the relative percentages of each language measure by gender. Figure S2 may be interpreted as another way of demonstrating that, while differences do exist between men and women in our data, most differences are relatively small. Of the greatest note, men used more prepositions (Cohen's *d* = .17), whereas women used higher rates of language consistent with depression and emotional upheavals; namely, negative emotion words (broadly defined; *d* = .16), anxiety words specifically (*d* = .15), and first-person singular pronouns (*d* = .14).

Figure S2

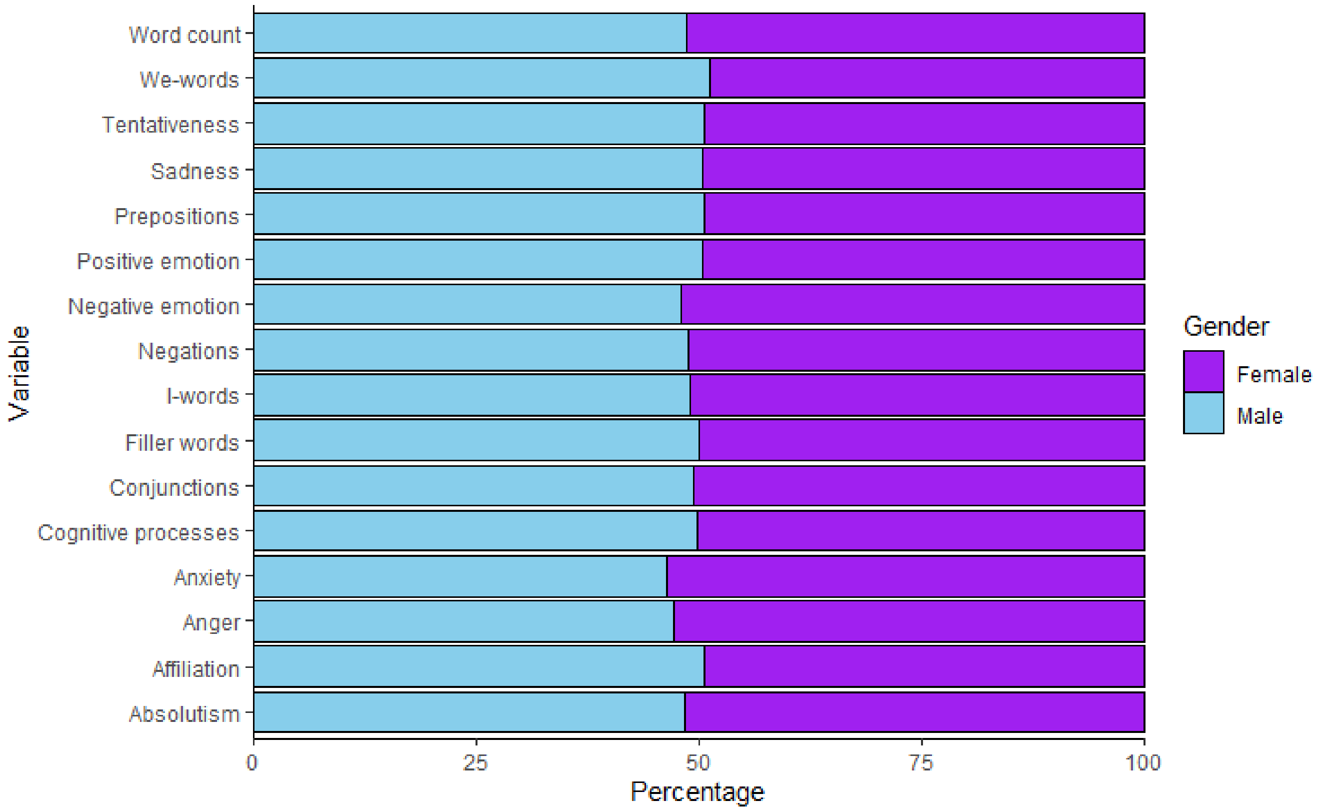

Differences in Language Measures Between Men and Women Discussing Their Relationships (Relative Percentages)

Supplementary Materials C:

Automated Detection of Theme Presence/Absence

Simply described, our goal was to only classify those submissions as containing each theme when there was clear evidence for a significant appearance of clusters of words related that theme were contained within the submission. For example, it would be inappropriate to classify a submission as containing the housework theme simply because the word “clean” appears within the text — the author may be referring to a “clean breakup” or a “clean slate” or some other sense of the word. However, if a submission contains several housework-related words, such as *clean, chore, vacuum, dishes*, and so on, we can be more confident that this submission is on-topic for this theme. Statistically, then, our goal was to establish a “noise floor” — a numerical threshold that would differentiate which texts contained an errant word or two that may or may not be related to a particular MEM theme versus texts that contained a sufficient number of theme-relevant words to be classified as containing that theme.

Automatic theme recognition was performed by quantifying the relative frequency of theme-related words in each submission, then comparing these values against theme-specific noise floors identified using the At Most One Change (AMOC) change point detection algorithm (see Killick & Eckley, 2014). Any submission scoring above each floor for any given theme was classified as containing that particular theme. Figure S3 provides an example of how the detection process with AMOC operates for the housework theme. MEM themes have a theoretical boundary from -100 to +100, with most texts scoring in the region around 0 (indicating an absence of a given theme). Via the AMOC algorithm, we establish 2 changepoints: the point at which any given text trends upwards *toward* zero, and the point at which any given text trends upwards *away from* zero; it is this latter changepoint that is of interest in the current context for each theme. We note here that this method — like all statistical methods for changepoint detection — is not perfect and, in some cases, may skew towards liberal or conservative inclusion points relative to what a human coder may judge. Rather, we emphasize that this method was used to heuristically identify texts that were likely to contain each MEM-derived theme. Figure S4 illustrates the distribution of MEM theme presence across our sample, with numbers along the X-axis reflecting the number of themes detected within any given submission.

For additional depiction of what each MEM theme reflects, as well as what types of content is captured under each theme, we present illustrative examples in Table S1.

Figure S3

Use of the AMOC Algorithm to Establish a "Noise Floor" Above which Any Post would be Classified as Containing the Housework Theme

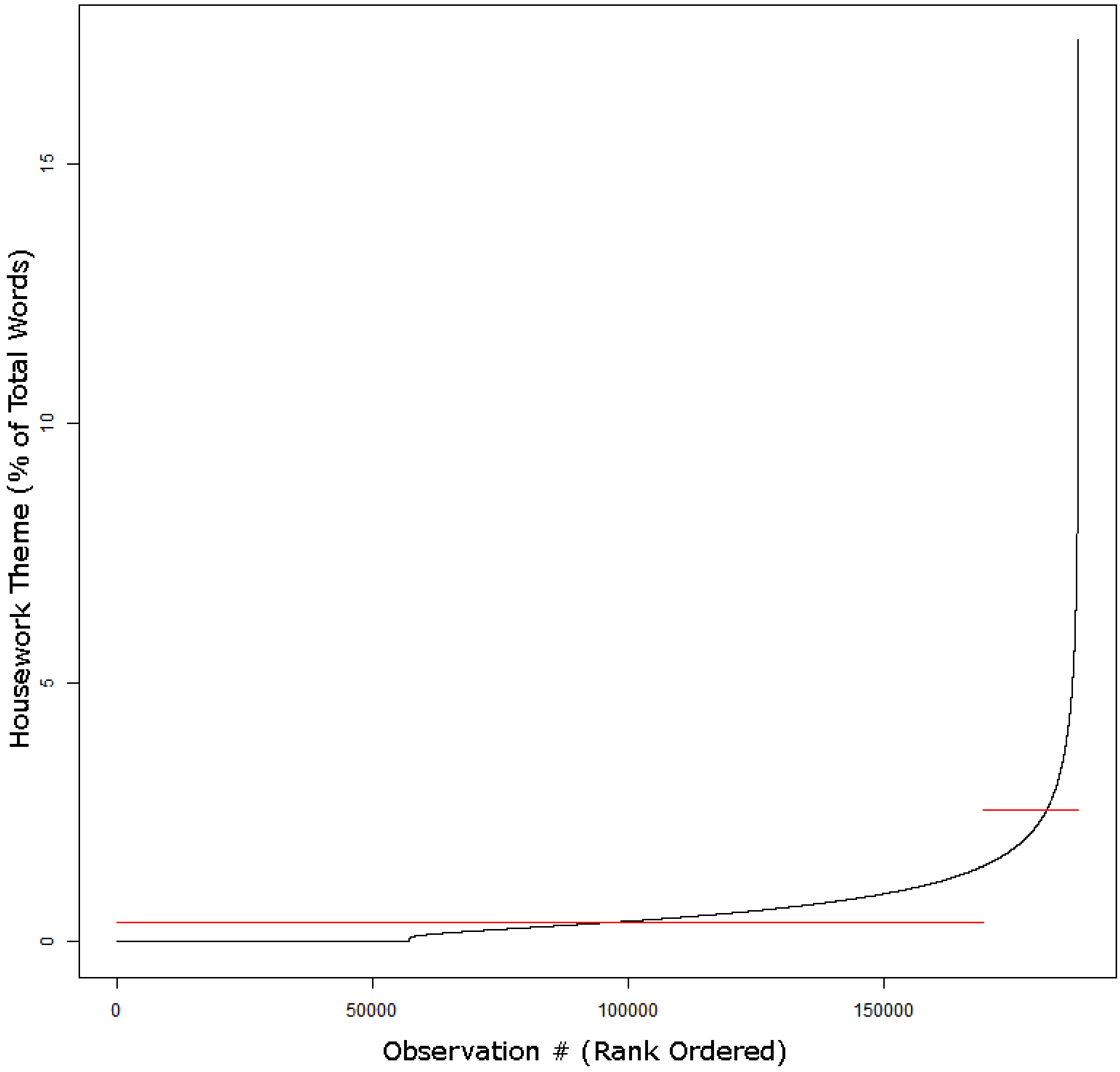

Figure S4

Distribution of the number of themes detected across r/relationships submissions, with a median of 3 found in our sample.

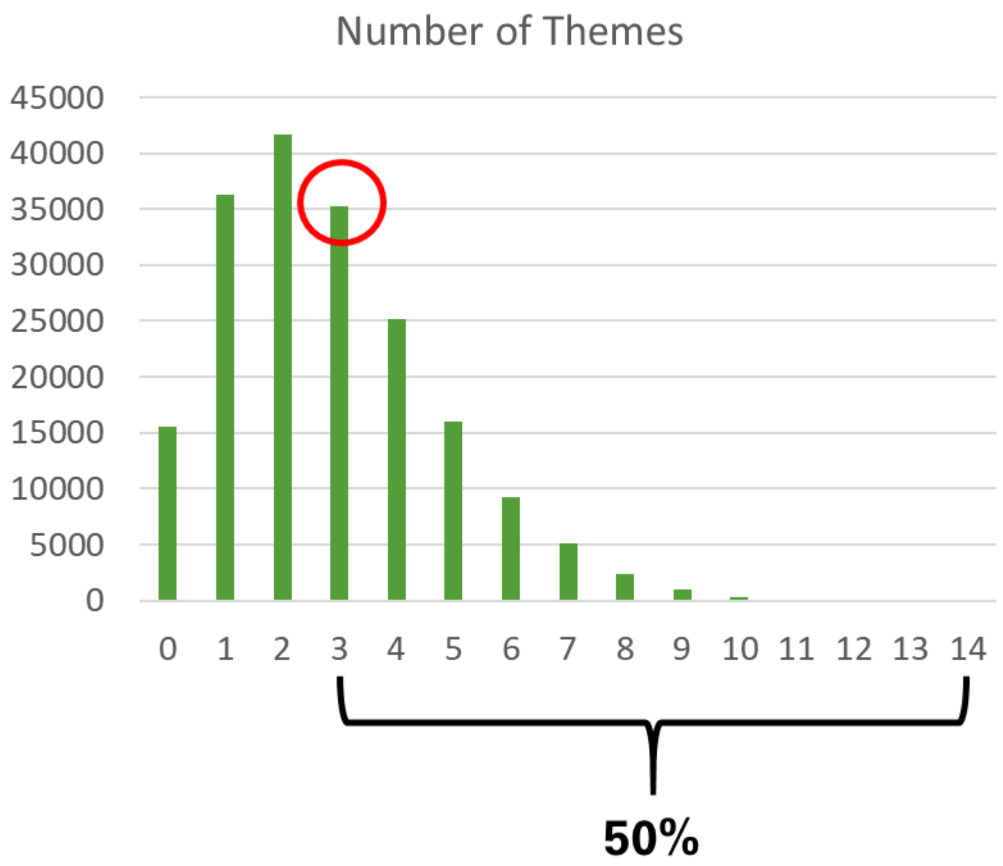

Table S1

Illustrative examples of MEM themes.

Theme	Example Words	Example
Heartache	Heart, Break, Hurt	I am having trouble dealing with heartbreak and still being friends with ex/friend/coworker.
Communication	Discuss, Express, Conversation	Girl ignored me for month, suddenly texted 'Hey'. Conversations keep getting nowhere.
Shared Feelings	Told, Upset, Feeling	I said I was sorry, but I couldn't leave until our agreed upon time… He's livid and says that he gave up his work out so I wouldn't be waiting
Time	Morning, Friday, Hour	I asked if 5:45 would work. He said he wanted to go to the gym after work, and that 6:15 would be better. I said ok no problem. At 5:40 I notice I have a few missed calls from him about 10 minutes ago.
Dating	Date, Casual, Hook-up	We started dating casually. I leave in about a week. Today we were hanging out and she broke down into tears. She says she can't take it because she is in love with me and that she doesn't think I feel the same way about her.
Personal Qualities	Cool, Nice, Funny	She's smart, funny, very sweet, understanding and supportive.
Trust Issues	Trust, Snoop, Cheat	What really gets my radar screaming is that when she returned home, I grabbed her phone while she was asleep to see if there was anything I had missed. The conversation had been deleted in her phone. From what I could tell, none of the other text threads had been deleted, and this is going back years.

Intimacy	Smile, Cuddle, Touch	I'm confused if he even likes me. He'll move to the floor so I don't cuddle him on the couch, is tired of sex half the time and mentioned it to him and now he seems like he just does it like he has too.
Partying	Party, Drunk, Invite	He started going to this bar … every single weekend, and every weekend he'd get drunk and party super late.
Abuse	Abusive, Threaten, Control	She cheated on me multiple times, mentally abused me … and continues to blame everything on me and refuse to give me my belongings (worth in the $1000s) for the past 6 months and threaten to burn them.
Distance	Move, Travel, Long-Distance	Our relationship is not without issues, no one's really is. Most of them stem from the fact that we go to university nearly six hours apart. We see each other every other weekend, and during breaks, but it still takes its toll. I think it's especially hard for her, as she had been in a previous [long-distance relationship] before we started dating.
Wedding	Marriage, Marry, Wedding	I am feeling pretty glum about my wedding, and I feel so bad about how it's affecting my fiancé.
Career	Job, Career, Company	I gave up my job nearly 6 years ago because it was apparently cheaper for us to have me at home. We live where he wants to live. Where it's convenient for him. I am so desperate to return to study and establish a career, and he assures me I can...as long as it fits in with the kids schedule.
Finances	Money, Pay, Debt	I spent all of my bonus without telling my wife. We disagree over whether my bonus is mine alone to spend, although we do combine other expenses. Am I wrong for thinking I should be able to

		spend my bonus how I want and that I didn't do anything wrong here?
Family/Parenting	Child, Pregnancy, Parent	I am very concerned having my wife be so stressed out during her pregnancy. This is my first child, her 3rd, and I do not want a miscarriage.
Mental Health Issues	Depression, Diagnose, Therapy	Girlfriend is very different from me and I might want to break up for various reasons. However I do love her, and because she suffers from anxiety and depression I'd feel so guilty for leaving her at a time like this
School	School, College, Semester	After moving here, she attended school for a few months but then stopped attending. No matter how much I kept urging her to sign up for her next semester classes, she just kept putting it off.
Hobbies	Video Game, Music, Sport	He never looks at job listings he just plays video games all day.
Religion	Religious, Belief, Church	We both want to continue dating, but she feels she needs someone who is a Christian like she is, and so has decided that we should not be together. I have no faith.
Housework	Cleaning, Laundry, Cooking	I enjoy having a clean living space, saving money, cooking healthy meals, and drinking/smoking weed occasionally. My partner is the opposite: he’s messy, spends all of his earnings, and daily enjoys fast food, alcohol, and weed
Sex	Sex, Masturbate, Porn	The idea that it takes effort for him to want to have sex with me yet he gets so turned on by other women's pictures really upsets me and crushes my self-esteem.

Language	English, Native, Language	The issue of me learning her native language (Russian) is causing constant tension and argument.
Body Weight	Lose-Weight, Overweight, Diet	Boyfriend of nearly 3 years has gained a lot of weight very quickly and I am starting to find him very unattractive
Substance Use	Drinking, Drug, Addict	I am afraid my girlfriend is going to relapse on drugs. What do I do to help her?
Romantic Gestures	Thoughtful, Gift, Celebrate	Most of the time he is honestly perfect. Understanding, loving, really really romantic and thoughtful etc. And I think that is why I am struggling so much with it, because it's a polar opposite to what I am used to.

Supplementary Materials D:

Gender Differences in MEM Theme Use

In the main body of the manuscript, we presented all gender difference analyses in MEM themes in the form of boxplots to provide an easy-to-navigate overview of the findings and to illustrate the general similarities between men and women. Here, we provide a more thorough account of the statistical analyses in the form of Table S2, below.

Importantly, note that while the effect sizes may be considered “small” using traditional interpretation guidelines, there today exists a common consensus that the size of an effect is not a meaningful indicator of its relative “importance” for several reasons. First, traditional social science research has relied on small sample sizes, resulting in 1) the need for an effect to be “large” to be detectable, 2) an inaccurate fixation on large effect sizes as markers of importance, and 3) gross over-estimates of common effect sizes, leading in large part to the current “replication crisis” (see, e.g., Anderson & Maxwell, 2017; Button et al., 2013). Rather, it is now fairly well-understood that small effects can be particularly important when they 1) occur in non-trivial contexts (such as in the context of real-world help-seeking and relationship problems, rather than an artificial lab study), 2) challenge existing theory and assumptions (as many of our current effects do), and/or 3) can have large cumulative consequences. For additional reading, we recommend Cortina and Landis (2009) and Matz et al. (2017).

Table S2

Gender Differences in the Relative Centrality of MEM Themes Discussed (N = 147,796)

Theme	Mean *(SD)* Men (*N* = 80,722)	Mean *(SD)* Women (*N* = 67,074)	*t*	*d*	95% CI
Heartache	14.79 (2.96)	14.25 (2.87)	35.63***	.19	.51 – .57
Communication	11.43 (2.64)	11.32 (2.57)	7.62***	.04	.08 – .13
Shared Feelings	10.56 (2.42)	10.71 (2.40)	-11.28***	.06	-.17 – -.12
Time	6.79 (2.53)	6.61 (2.45)	13.33***	.07	.15 – .20
Dating	6.27 (2.61)	5.64 (2.51)	46.63***	.25	.60 – .65
Personal Qualities	5.30 (2.01)	5.06 (1.87)	23.95***	.12	.22 – .26
Trust Issues	4.62 (2.32)	4.43 (2.34)	15.77***	.08	.17 – .22
Intimacy	4.01 (1.98)	3.88 (1.81)	12.85***	.07	.11 – .15
Partying	3.58 (1.80)	3.34 (1.70)	26.61***	.14	.22 – .26
Abuse	3.24 (1.60)	3.46 (1.72)	-25.26***	.13	-.24 – -.20
Distance	2.90 (1.77)	3.12 (1.88)	-22.75***	.12	-.24 – -.20
Wedding	2.82 (1.40)	2.72 (1.39)	14.09***	.07	.09 – .12
Career	1.75 (1.46)	1.84 (1.56)	-11.31***	.06	-.11 – -.07
Finances	1.52 (1.51)	1.77 (1.71)	-29.28***	.15	-.27 – -.23
Family/Parenting	1.50 (1.46)	1.64 (1.51)	-18.18***	.09	-.16 – -.13

Mental Health Issues	1.25 (1.08)	1.34 (1.11)	-15.90***	.08	-.10 – -.08
School	1.38 (1.61)	0.75 (1.38)	80.76***	.42	.61 – .64
Hobbies	0.86 (1.38)	0.82 (1.37)	4.91***	.03	.02 – .05
Religion	0.63 (0.90)	0.69 (0.94)	-12.60***	.07	-.07 – -.05
Housework	0.54 (0.75)	0.69 (0.94)	-33.44***	.18	-.16 – -.14
Sex	0.51 (1.20)	0.62 (1.22)	-16.89***	.09	-.12 – -.09
Language	0.65 (1.06)	0.43 (0.89)	43.08***	.22	.21 – .23
Body Weight	0.39 (0.65)	0.43 (0.71)	-10.32***	.06	-.04 – -.03
Substance Use	0.17 (0.41)	0.19 (0.48)	-8.99***	.04	-.03 – -.02
Romantic Gestures	0.01 (0.77)	0.01 (0.87)	-1.97*	.01	-.02 – -.00

$^{*}p<.05$, $^{***}p<.001$.

Note. Means refer to percentages of MEM themes discussed within each *r/relationships* submission. CI = confidence interval.

Supplementary References

Anderson, S. F., & Maxwell, S. E. (2017). Addressing the “replication crisis”: Using original studies to design replication studies with appropriate statistical power. *Multivariate Behavioral Research*, *52*(3), 305–324. https://doi.org/10.1080/00273171.2017.1289361

Button, K. S., Ioannidis, J. P. A., Mokrysz, C., Nosek, B. A., Flint, J., Robinson, E. S. J., & Munafò, M. R. (2013). Power failure: Why small sample size undermines the reliability of neuroscience. *Nature Reviews Neuroscience*, *14*(5), 365–376. https://doi.org/10.1038/nrn3475

Cortina, J. M., & Landis, R. S. (2009). When small effect sizes tell a big story, and when large effect sizes don’t. In *Statistical and methodological myths and urban legends: Doctrine, verity and fable in the organizational and social sciences* (pp. 287–308). Routledge/Taylor & Francis Group.

Killick, R., & Eckley, I. A. (2014). Changepoint: An R package for changepoint analysis. *Journal of Statistical Software*, *58*(1), 1–19. https://doi.org/10.18637/jss.v058.i03

Matz, S. C., Gladstone, J. J., & Stillwell, D. J. (2017). In a world of big data, small effects can still matter. *Psychological Science*. https://doi.org/10.1177/0956797617697445
